# Therapist perspectives on telehealth-based virtual reality exposure therapy

**DOI:** 10.1007/s10055-024-00956-7

**Published:** 2024-03-08

**Authors:** Triton Ong, Julia Ivanova, Hiral Soni, Hattie Wilczewski, Janelle Barrera, Mollie Cummins, Brandon M. Welch, Brian E. Bunnell

**Affiliations:** 1 Doxy.Me Research, Doxy.Me Inc, Rochester, NY, USA; 2 Department of Psychiatry and Behavioral Neurosciences, Morsani College of Medicine, University of South Florida, Tampa, FL, USA; 3 College of Nursing and Department of Biomedical Informatics, University of Utah, Salt Lake City, UT, USA; 4 Biomedical Informatics Center, Medical University of South Carolina, Public Health and Sciences, Charleston, SC, USA

**Keywords:** Virtual reality, Exposure therapy, Telehealth, Mental health, Clinical practice

## Abstract

Virtual reality (VR) can enhance mental health care. In particular, the effectiveness of VR-based exposure therapy (VRET) has been well-demonstrated for treatment of anxiety disorders. However, most applications of VRET remain localized to clinic spaces. We aimed to explore mental health therapists’ perceptions of telehealth-based VRET (tele-VRET) by conducting semi-structured, qualitative interviews with 18 telemental health therapists between October and December 2022. Interview topics included telehealth experiences, exposure therapy over telehealth, previous experiences with VR, and perspectives on tele-VRET. Therapists described how telehealth reduced barriers (88.9%, 16/18), enhanced therapy (61.1%, 11/18), and improved access to clients (38.9%, 7/18), but entailed problems with technology (61.1%, 11/18), uncontrolled settings (55.6%, 10/18), and communication difficulties (50%, 9/18). Therapists adapted exposure therapy to telehealth by using online resources (66.7%, 12/18), preparing client expectations (55.6%, 10/18), and adjusting workflows (27.8%, 5/18). Most therapists had used VR before (72.2%, 13/18) and had positive impressions of VR (55.6%, 10/18), but none had used VR clinically. In response to tele-VRET, therapists requested interactive session activities (77.8%, 14/18) and customizable interventions components (55.6%, 10/18). Concerns about tele-VRET included risks with certain clients (77.8%, 14/18), costs (50%, 9/18), side effects and privacy (22.2%, 4/18), and inappropriateness for specific forms of exposure therapy (16.7%, 3/18). These results reveal how combining telehealth and VRET may expand therapeutic options for mental healthcare providers and can help inform collaborative development of immersive health technologies.

## 1 Introduction

The World Health Organization reported a 25% global increase in anxiety and depressive symptoms during the COVID-19 pandemic (WHO 2022). It is estimated that 1 in 3 people are at risk for serious mental health disorders related to anxiety, stress, and phobias (Panchal et al. 2023). Therapists face unprecedented burnout and turnover with rapidly accelerating demand for mental health services (Van Hoy and Rzeszutek 2022). Technological solutions are needed to extend clinicians’ capabilities and meet growing demands in mental health care (National Academy of Medicine 2019).

Telehealth—the delivery of health care using remote communication technologies like phones, videoconferencing, and the internet—is one such solution that has been demonstrated to make therapy more accessible, less stigmatized, more convenient, and more cost effective (Barnett et al. 2021; Madigan et al. 2021; Marcu et al. 2022). Since the COVID-19 pandemic, mental health care has made up the majority (57.9%) of telehealth use (Trilliant Health 2022). Telehealth has produced clinical results noninferior to traditional in-person therapy for eating disorders, depression, schizophrenia, anxiety, and trauma (Batastini et al. 2021; Greenwood et al. 2022). While telehealth for mental health care (i.e., telemental health care) has been embraced broadly, some therapists and clients also report feeling less capable of expressing themselves over phone or video calls (Aviram and Nadan 2023; Connolly et al. 2022; Cowan et al. 2019), which may negatively impact therapeutic relationships (Aafjes-Van Doorn et al. 2023).

Exposure therapy is the gold standard treatment for anxiety disorders such as specific phobia, social anxiety, posttraumatic stress disorder (PTSD), and obsessive–compulsive disorder (OCD) (Steinman et al. 2016). However, it can be challenging to recreate anxiety-related situations or handle such stimuli in a clinic office, making most therapists unlikely to use exposure therapy despite knowing it could benefit their clients (Pittig et al. 2019). It is common for people with anxiety to develop patterns of avoidance, including avoiding therapy or therapists who may agitate their anxiety in the course of treatment (Dymond 2019). For example, military veterans with severe PTSD can find it unbearable to even discuss their traumatic experiences, making exposure therapy too time-intensive and emotionally straining to consider (McLean et al. 2022). Virtual reality (VR) is a promising technology that can enhance the treatment of anxiety and related disorders (Emmelkamp and Meyerbröker 2021). VR combines interactive computer simulations and encompassing display systems to create immersive therapeutic experiences, and has been used most frequently in the treatment of anxiety disorders (Cieślik et al. 2020). Using VR-based exposure therapy (VRET), researchers were able to produce large clinical improvements compared to non-VR psychotherapy for veterans with treatment-resistant PTSD (van Gelderen et al. 2020). Research has shown that exposure therapy using VR simulations can help patients overcome avoidance patterns and approach therapy more comfortably, resulting in stronger treatment engagement, improved comfort and satisfaction, and clinical outcomes equivalent to or sometimes better than in-person exposure (Carl et al. 2019; Deng et al. 2019; Parsons and Rizzo 2008). While research has demonstrated the clinical efficacy of VRET, more recent studies have focused on challenges to real-world implementation of VRET such as limited clinic space, reliance on in-person assessment, and the absence of integrated clinical solutions (Boeldt et al. 2019; Wray and Emery 2022).

There is growing potential to combine VR and telehealth to expand the ways people can receive mental health therapy (Ong et al. 2022). In recent years, everyday use of VR has grown to include internet-based interactions to foster relationships, meet new people, and engage in creative forms of self-exploration (Barreda-Ángeles and Hartmann 2022). Many VR enthusiasts have used a popular social VR platform called VRChat to reduce negative thoughts, alleviate isolation, increase opportunity and ease of socializing, and explore other forms of self-directed mental health care during the pandemic (Deighan et al. 2023; Kelley 2021; Sykownik et al. 2021; Zamanifard and Robb 2023). However, only 9% of people who bought VR during the pandemic did so for telehealth purposes (Ball et al. 2021). Despite the promise of VR for telemental health care, most VR is used currently for personal entertainment and not for formal therapy.

Integrating the convenience of telehealth and the flexibility of VR can empower therapists with innovative solutions and improve client access to evidence-based treatments such as exposure therapy. To our knowledge, previous studies have examined therapist opinions on clinic-based VRET (Twamley et al. 2022), but no studies have examined therapist perspectives on VRET delivered via telehealth (i.e., tele-VRET). To understand end-user perspectives and inform the design of highly usable and effective tele-VRET, it is critical that these solutions be co-designed in direct collaboration with mental health therapists and clients (Bevan Jones et al. 2020; Chung et al. 2022). Toward that end, we conducted semi-structured interviews with telemental health therapists to investigate their past experiences, current opinions, and future perspectives on tele-VR for anxiety and related disorders.

## 2 Method

### 2.1 Study design

We used a qualitative design that included semi-structured, qualitative, individual interviews with therapists.

### 2.2 Participants and recruitment

We recruited therapists from TelehealthEngage, a research registry of more than 5,000 independent healthcare professionals registered with Doxy.me, a commercial telemedicine software provider. Therapists were invited to participate in the study if they met the following inclusion criteria: (1) spoke English fluently, (2) were an actively practicing mental health therapist at the time of the study (October–December 2022), (3) used telehealth to provide mental health care at the time of the study, and (4) had previously used telehealth to conduct exposure therapy. Therapists were notified they would be compensated with a $75 USD eGift card upon completion of their interview.

### 2.3 Procedures

**Fig. 1:**
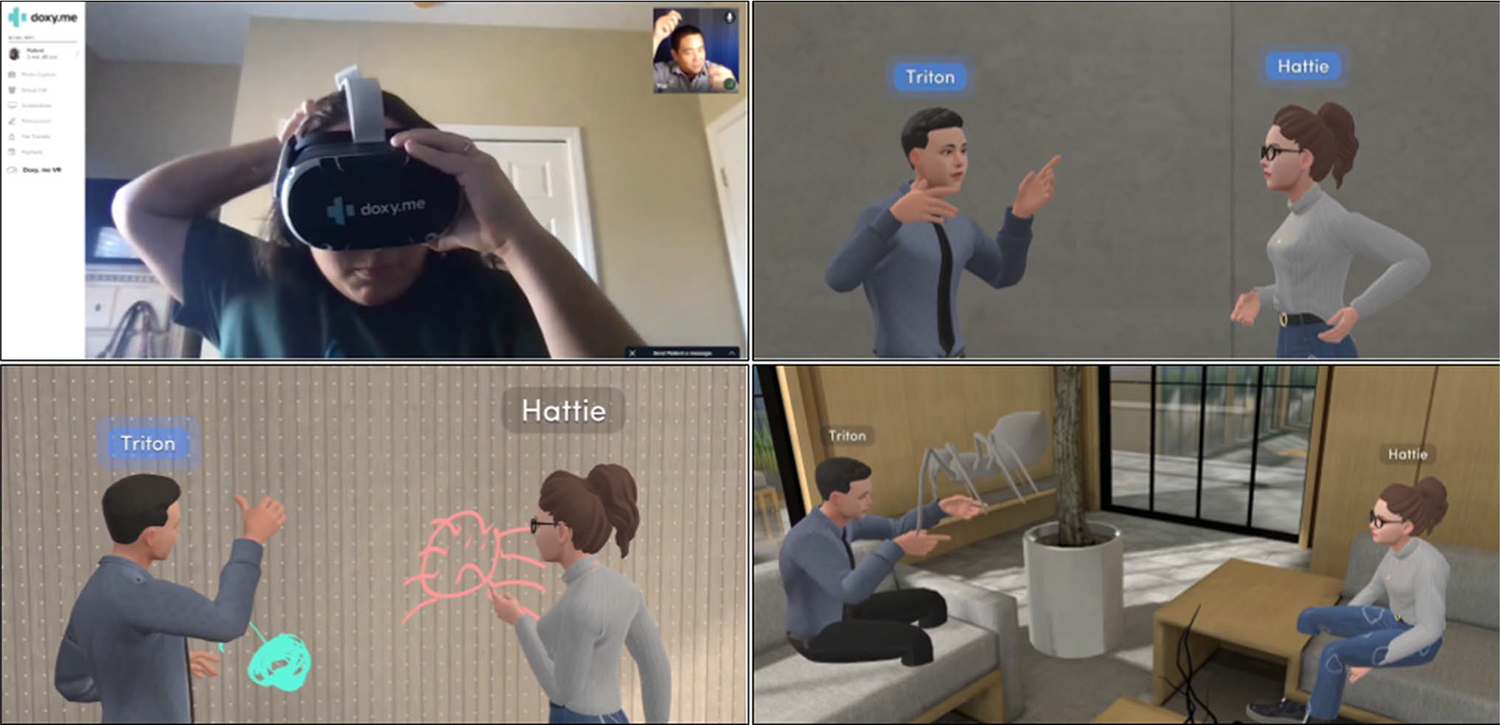
Screenshots from the tele-VRET video. Therapist showing a client how to use VR during a telehealth call (top left). Therapist and client talking and gesturing remotely in VR (top right). Therapist and client drawing spiders in VR (lower left). Therapist and client interacting with a simulated spider in VR (lower right)

Study sessions consisted of a single, one-on-one, recorded, one hour long, online interview using a secure version of Google Meet. The first author conducted the interviews using a semi-structured guide (Appendix 1). Interviews proceeded in five sections. Section 1 consisted of a discussion of informed consent, which detailed our research team’s exclusive access to deidentified interview transcripts, and five basic demographic questions (i.e., specialty, age, sex, ethnicity, race). Section 2 included questions about therapists’ general experiences with telehealth. Section 3 focused specifically on telehealth for exposure therapy. Section 4 involved discussion of therapists’ prior experiences with VR and perceptions of VR for mental health therapy. At the end of Sect. 4 and before the fifth, the researcher played a 1 min and 35 s video describing tele-VRET (Fig. 1). Finally, Sect. 5 was a discussion of therapists’ impressions, concerns, needs, and wants regarding tele-VRET.

During each interview, we reminded participants their compensation was contingent on completion of the interview only and their candid, direct feedback would be most valuable to the research. Study procedures were reviewed and approved as exempt by the Institutional Review Board of the University of South Florida (IRB003548) prior to the conduct of this research.

### 2.4 Data analysis

Interview audio recordings were transcribed into text using Dovetail and then analyzed using MAXQDA 2022. The first author (an Asian-American male and behavior scientist experienced with VR therapy research) led thematic analysis of the transcribed interviews using meaningful phrases as the coding unit (Braun and Clarke 2012, 2019). The researcher used repetitions, similarities and differences, cutting and sorting, and metacoding techniques to identify and organize themes over the course of three iterations (Bernard et al. 2016; Nowell et al. 2017). Therapist statements within respective codes were presented as percentages of total participants. We aimed to identify emergent themes related to the four main sections of the interview (i.e., telehealth, exposure therapy over telehealth, prior experience with VR, and perspectives on tele-VRET). Themes and operational definitions were honed across three iterations, upon which the second author (a Caucasian female immigrant and medical anthropologist experienced in psychology and biomedical informatics) reviewed the codebook and all codes. We did not conduct intercoder reliability as only the first author was experienced in VR therapy research. Instead, discrepancies between the first and second authors’ interpretations of the codes and codebook were resolved through discussion until consensus to ensure consistency and accuracy in the qualitative method (Campbell et al. 2013; Raskind et al. 2019).

## 3 Results

### 3.1 Participant and practice characteristics

A total of 18 therapists were interviewed. Most participants identified as female (89%; 16 female, 2 male), white (72.2%; 13 white, 3 black, 1 Asian, 1 multiracial), non-Hispanic (94.4%; 17 non-Hispanic, 1 Hispanic), and middle age (M = 44 years, SD = 10.13, range 27–71). Professional titles included psychologist (39%, 7/18), mental health counselor (28%, 5/18), social worker (16.5%, 3/18), and marriage and family therapist (MFT; 16.5%, 3/18). Main disorders treated were anxiety (100%, 18/18) and trauma (100%, 18/18), with some providing therapy for personality disorders (61%, 11/18), depression (39%, 7/18), and substance abuse (5%, 1/18). Primary therapeutic approaches included cognitive behavioral therapy (50%, 9/18), dialectical behavioral therapy (28%, 5/18), and acceptance and commitment therapy (22%, 4/18). All participants treated adults (100%, 18/18) as their primary clientele, with some also providing services for children (22%, 4/18), families (5%, 1/18), or individuals across the lifespan (5%, 1/18). Some therapists (33%, 6/18) reported adopting telehealth between 2013 and several months before the COVID-19 pandemic, but most (67%, 12/18) started providing telemental health care after the pandemic had begun.

### 3.2 Benefits of telemental health care

We asked therapists about their history, practices, and preferences with telemental health care. In response, therapists described how telehealth made it easier for their clients to receive mental health services, reduced the effort to conduct in-session therapeutic exercises, and provided a more holistic view into clients’ everyday lives.

#### 3.2.1 Reduced barriers for clients

Nearly all participants (88.9%, 16/18) reported ways in which telehealth made it easier, more convenient, and more comfortable for clients to engage in therapy. Reduced travel burden was the most commonly reported way that telehealth reduced barriers (61.1%, 11/18). Since clients could access telehealth from personal and mobile devices, they experienced less need to alter their daily schedules for travel and avoided the stress of navigating traffic before and after a session. Therapies that required detailed scheduling and coordination when done in-person, such as support groups, were reported to be more accessible for clients over telehealth as they could join from home without the need for childcare. Some therapists (16.7%, 3/18) reported how some clients seemed concerned about stigma (e.g., being recognized in an in-person waiting room) and that telehealth helped provide a more private care experience. Some therapists (16.7%, 3/18) also cited continuity of care throughout the COVID-19 pandemic as a benefit of telemental health services.

They can sneak out to their car on their lunch break. It just makes it so the people that might have had more barriers to therapy can more easily access it now.

(Therapist 14)

#### 3.2.2 Telehealth enhanced aspects of mental health practice

Most therapists (61.1%, 11/18) reported that telehealth made it easier to conduct some in-session mental health practices. Therapists described how the remote format of telehealth allowed them to feel more confident when helping clients confront sensitive issues and challenging beliefs.

I can be a little more pushy or assertive because there isn’t as much tension over the computer as you might feel in the room. Sometimes I’ll notice that I’ll get a little flushed or I’m uncomfortable or it feels really intense [in-person] and I think that gets diluted over the computer. You can go a little further with certain lines of questioning or confrontation.

(Therapist 10)

Therapists also enjoyed how telehealth allowed them to simplify clinical workflows. While in-person therapy could involve handling a variety of devices within a session, some therapists (16.7%, 3/18) found that using telehealth allowed them to access a variety of functions from their computer to use features like assessments, screen sharing, chat boxes, and video. Some therapists (16.7%, 3/18) also reported that providing telehealth from home allowed them to better enjoy the flow of their work with more restful breaks and comfortable spaces.

I like that I’m wearing my slippers right now. I like how I have more time to do other things instead of the commute and it’s just easier than having to pack a lunch.

(Therapist 14)

Some therapists (16.7%, 3/18) reported that offering telemental health services improved business aspects of their practice. Telehealth allowed therapists to increase their case-loads with larger client pools from greater distances, expand their operational hours into different time zones, reduce or eliminate rented clinic offices, reduce expenses enough to justify taking on additional staff or supervisees, and more flexibility to prevent late or canceled sessions.

I’m able to practice with so many other clients that would normally have to travel out to meet with me. Now I’m able to get them in a couple of time zones. I’m able to push back the time in my office a little bit at night also. There’s a lot of flexibility there. None of this would’ve been possible without telehealth.

(Therapist 11)

#### 3.2.3 Seeing naturalistic settings

Some therapists (38.9%, 7/18) reported appreciating how telehealth let them see clients outside the clinic. Clients’ presentations during telehealth sessions provided valuable insights and opportunities to advocate for client well-being, self-care, and living situations which may have gone undetected during an in-person session.

It gives you a view into people’s world that you might not otherwise get. I had someone connect to a session from their closet under a blanket and I was like, “what’s going on?” We ended up calling the police and the abuser was removed from the home, which probably wouldn’t have happened had they not [joined via telehealth], right?

(Therapist 4)

One therapist reported how telehealth allowed them to share their own settings to make a stronger interpersonal connection with a client.

One time my dog walked up and was sitting in the frame. Typically I closed the door so that the dog can’t get into this room. But for some reason I left the door open that day, and it was just happenstance that it helped me make a connection with my seven year old client because the client had a dog. So at that point, I guess I became more human to the client. So what I thought was gonna be a disruption was actually helpful.

(Therapist 13)

### 3.3 Limitations of telemental health care

While therapists were positive about telehealth overall, they reported experiencing disruptive technical issues, feeling they had less control over remote session settings, and that communication over telehealth was not as rich as the in-person experience.

#### 3.3.1 Technology problems

Most therapists (61.1%, 11/18) reported encountering technical problems with telehealth. Internet issues were the primary concern due to weak signal, low bandwidth, or inconsistent connectivity. These internet problems could disrupt audio and video signals and impact the progress of therapy sessions.

I hate when I’m in the middle of a processing session and it glitches and I need them to repeat something they said. When we’re processing, it feels like the internet is messing with my ability to be as present and attuned as I’d like to be. If it actually freezes, I’m horrified.

(Therapist 14)

A few therapists (11.1%, 2/18) also described restrictive organizational policies or security programs that limited their access to online content. These technical restrictions prevented therapists from using websites, search engines, or YouTube videos as part of their telehealth exposure practices.

Normally in [prolonged exposure], I might pull out something in session. I have to do it on my phone oftentimes because my [work] computer will not let us access YouTube. I might pull up a sound that is particularly triggering and we’re gonna sit here and play it until it gets darn boring. I haven’t done that sort of thing by telehealth. The crazy thing is, [clinic admins] send us links like, “we’ve just produced this lovely YouTube video, check it out.” And I’m like, “how are we checking that out?” I’ll click on that link, but it’s going to say, “access forbidden.”

(Therapist 4)

#### 3.3.2 Less formal session environments

Most therapists (55.6%, 10/18) reported feeling they had less control over telehealth session arrangements. When meeting in-person, therapists dedicated substantial efforts to create safe and comforting physical spaces. Over telehealth, however, therapists reported fewer options to personalize session settings and expressed concern over their inability to intervene physically if necessary.

When I was in person, I always had spaces that were specifically structured to be welcoming. [Telehealth] is different, right? There’s a lot more effort on my end.

(Therapist 9)

You just never fully know. Even if someone looks really high functioning and well resourced, every once in a while something severe can happen. I can’t intervene [over telehealth] in the same physical ways as I can in person. I am way more cautious and I ask a lot of different questions about what’s going on in their environment.

(Therapist 14)

Some therapists (11.1%, 2/18) described new distractions in their telehealth practices. To their dismay, therapists noticed how telehealth made it easy to stealthily direct attention away from a client to check notifications or engage in unrelated administrative tasks during a session. Telehealth also introduced the need to set expectations as some therapists (16.7%, 3/18) had clients join sessions inappropriately while in line at a grocery store, at another therapists’ office, driving in traffic, or while undressed in bed.

We probably could improve our orientation to people about what’s acceptable. I’m on a text thread with a lot of my colleagues and we’ve all had that one moment where we’re like, “how could someone think this is okay?” It’s just probably about reestablishing protocol in etiquette for a really new thing that none of us were doing a couple years ago.

(Therapist 10)

#### 3.3.3 Communication difficulties

Half of the therapists (50%, 9/18) reported that telehealth limited their access to some forms of nonverbal communication. Reduced visibility of body language meant that some therapists felt they could not fully assess client affect. Subtle signs of distress, such as tapping feet or fidgeting hands, were easily detectable during in-person sessions. However, webcam and smartphone setups did not display such events over telehealth. Therapists’ own nonverbal communication was also limited over telehealth, as some clients interpreted meaningless changes in therapist gaze or posture as discomfort or disapproval.

The only thing that’s missing from telehealth is the interpersonal thing. I think that telehealth only gives you maybe 70% of a person. You miss a lot of non-verbals. You can’t see the person’s whole body. Are they fiddling with their fingers? Are they wiggling their feet? You only get from [shoulders] up and it would be very weird for you to ask the person, “can you pan your camera all the way back so I can see your whole body?” So you kind of just take the face and not the rest. But that’s just the nature of what it is to be on camera. You just miss that richness of the full in person experience.

(Therapist 1)

Half the therapists (50%, 9/18) also described how some client symptoms could be misaligned with remote care. Anxiety therapy often overlapped with client concerns about online privacy, unauthorized recording, or surveillance by intelligence agencies. Telehealth was also described as risky for clients with covert symptoms. Clients with eating disorders, for example, could hide rapid weight change by framing or obscuring their body on a video call. Some therapists also reported the convenience and comfort of telehealth could be countertherapeutic for clients who would benefit from positive side effects of in-person care like grooming, getting dressed, and interacting with people outside the home.

I think sometimes [telehealth] can keep people isolated a little bit. I think people have a lot of fears since the pandemic and now, to go to therapy, they don’t have to go out and do other things. So sometimes [I need to] challenge people to get outside of their comfort zone and not just stay home just because it feels safer.

(Therapist 17)

### 3.4 Adaptations of exposure therapy over telehealth

After discussing telemental health in general, therapists were asked about how they used telehealth to provide exposure therapy. Therapists spoke frequently about the difficulty of providing exposure therapy in-person and how telehealth could involve new challenges and opportunities related to useful telehealth features and tools, the need to prepare clients in advance, and necessary adjustments to their workflows.

#### 3.4.1 Incorporating telehealth tools into exposure therapy

Therapists reported utilizing various telehealth features to enhance how they provided exposure therapy remotely. Many therapists (66.7%, 12/18) reported finding exposure stimuli in the form of videos or audio on *YouTube*. Most therapists (55.6%, 11/18) reported using telehealth to *transfer files* between themselves and their clients. Files transferred included informational pamphlets, clinical forms and questionnaires, worksheets, and client-generated media such as photos, voice recordings, or video. Many of these file transfer tools were built into the telehealth platforms or electronic health records, and some therapists used email as per client preference. Most therapists (55.6%, 10/18) reported *screen sharing* clinical documents, educational materials, and multimedia for exposures such as photos, videos, and websites that displayed strobing content designed for specific therapies such as eye movement desensitization and reprocessing. Half the therapists (50%, 9/18) reported directing clients to *informative websites* authored by reputable clinical organizations such as Barnes Hospital or Mayo Clinic. Some therapists (16.7%, 3/18) reported using *collaborative documents* to work with clients (e.g., Google Docs or digital white-boards). One therapist (6%, 1/18) reported recommending their clients try *smartphone apps* for meditation such as Calm, Headspace, and Insight Timer.

They’re bringing up all kinds of things on the platform that I can use as a clinician. They’ve got practice sessions with breathing and with assessments, and they’ve got a whiteboard. So now my children [clients] can draw and I can do all that stuff now. I can share my screen, I can play videos. It feels as if I can do more on telehealth than what I could do sitting face to face, because I don’t have to deal with pulling out a tablet or turning on the television to show a video.

(Therapist 13)

#### 3.4.2 Preparing clients expectations

Most therapists (55.6%, 10/18) reported making efforts to prepare clients for exposure therapy over telehealth. Therapists described the potentially stressful nature of exposure therapy with transparency, and how it could take several months to more than a year to achieve clinical success.

I consistently normalize how much exposure sucks, especially for trauma patients. It’s the worst treatment in the world. It’s so effective, but it’s the worst. Exposure sucks until it doesn’t and then it’s great.

(Therapist 15)

In addition to preparing client expectations, half the therapists (50%, 9/18) described dedicating time to building rapport and client competence as prerequisites to exposure therapy over telehealth.

Some of these clients come to me without even the capacity to tell their story because they’re so anxious about what they experienced, or they’re so afraid that I’m going to judge them. There’s a lot of shame or guilt around what has happened. A lot of times it’s really setting up the platform of them feeling comfortable.

(Therapist 13)

#### 3.4.3 Workload adjustments for telemental health care

Some therapists (27.8%, 5/18) described how pre-pandemic burnout compounded with mid-pandemic mental health surges into greater overall stress. Strategies to navigate this stress included sketches or shorthand instead of written session notes, limitations on new clientele (e.g., refusal to use telehealth for child clients due to distractibility), and shared expectations of patience while co-navigating challenges.

You never know what format you’re going to get [client data] in. If you can’t open the document, if it’s too big they can’t send it. Thank goodness for people who understand how to compress a PDF or know how to take screenshots. Or they actually just pick it up and show it to me on the screen and then I’m writing it down on the other side. I think everybody had a lot of patience with everybody. They’re frustrated and I’m frustrated, but we’re not really frustrated with each other.

(Therapist 1)

### 3.5 Previous experience and perceptions of VR in general

We asked therapists to discuss how they conceptualized VR, prior experiences using VR, overall impressions of VR, and what they had heard about VR for mental health care. For this discussion of their previous experiences with VR, we asked about their experiences with VR in general and did not ask about VRET until the proceeding section of the interview.

Therapists defined VR as consisting of three common components: an immersive experience (83.3%, 15/18), video games (61.1%, 11/18), and a head-mounted display (55.6%, 10/18).

I guess there could be multiple levels to it because in some sense even a standard video game has some sense of virtual reality to it. However, I think that we nowadays think of VR as something where your own body movements impact the place, impact the environment directly, right? On the Oculus, you have like these [controllers], the movements of them and of your hands. It’s not just, “hit the button.” It’s that sense in which your own body’s movements will affect the environment. And then because we have that feedback, then it makes the experience feel more realistic so that you then can have more engagement of your physical system, to trigger your vestibular system more successfully and that sort of stuff.

(Therapist 4)

Most therapists (72.2%, 13/18) had hands-on experience with VR while only some (27.8%, 5/18) had never tried VR. Of those who had tried VR, 8 (44%) tried VR in a professional setting such as conferences or in their institutions, and 8 (44%) tried VR in a casual setting such as theme parks or a friend’s house. One therapist tried VR for the first time when a client brought their headset into the office and showcased how they had been treating their own agoraphobia with a VR house building game and Beat Saber. Some therapists (33.3%, 6/18) reported personally owning a VR device; however, none used VR regularly as it was purchased for other members of their household (e.g., children).

I went over to [a colleague’s] house and he put these goggles on me. And I went to Paris and walked around and did all this cool stuff [in VR]. And he was talking to me about how this is so great for people who have to think about managing emotion and coping with situations that they’re not actually in. How do you simulate that? To me, it’s bringing so much more variety of experiences into the session. Historically, people who worked with anxiety would be like, “we’re going to get in your car and go driving.” Or, “I’m going to go on an airplane with you.” I think most people just don’t have that flexibility [without VR].

(Therapist 10)

When asked about their overall impressions of VR, most therapists (55.6%, 10/18) gave positive descriptions of captivating experiences and interests in clinical applications.

I think it can be a cool form of entertainment for some people. I think it can also be really valuable therapeutically in terms of treatment, especially for something like exposure. The more complicated or less common ones that are harder for people to actually do, like getting on a plane for fear of flying. They’re not going to get on a plane every day just to do exposures, right? So for something like that I could see VR being really helpful and just creating the same environment for them to actually do the exposures in, but not in real life.

(Therapist 16)

Neutral impressions of VR (33.3%, 6/18) included skepticism about its utility or general unfamiliarity.

I think there’s some really good stuff with virtual reality, but I still don’t think that it replaces the real thing. I can put a VR on and go for a walk in the woods, but I’m not going to get the health benefits of the fresh air and the UV, right?

(Therapist 13)

Negative impressions of VR (33.3%, 6/18) consisted of previous VR experiences that resulted in nausea, low perceived realism, and anticipation that VR would introduce barriers to communication.

First thing that comes to mind is the equipment, which I don’t know why, but it turns me off. Having that kind of equipment attached to your face, I don’t know. It feels like it’s a barrier.

(Therapist 12)

Therapists were generally knowledgeable about VR for mental health therapy and heard about it in professional contexts (72.2%, 13/18) at conferences, through colleagues, or in research articles. Some therapists heard about VR therapy casually (11.1%, 2/18) from a family member, news story, or through video games. Only three therapists (16.7%) reported no awareness of VR for mental health therapy. None of the therapists had tried VR in their mental health practices.

### 3.6 Perspectives on telehealth-based VRET

After viewing the video demonstration of a tele-VRET session, we asked therapists about their initial reactions to the proposed system and follow-up questions about how tele-VRET could meet the needs of their telehealth practices. Most therapists (66.7%, 12/18) expressed positive reactions about how VR could simplify exposure therapy practices and help telehealth clients feel their therapist’s presence in the healing experience.

I don’t like doing in-the-field type of work with [in-person] clients just logistically…so this seems like a way that I could do both. Kind of not both, clearly they’re not actually in the field but could do an actual exposure while they’re in the office and or at home without it being a lot of logistics to try to figure out.

(Therapist 5)

I like the fact that I could see their full body and they could see my full body. I liked the drawing on the wall. That was kind of interesting, it felt more collaborative than VR I’ve seen before where I had to imagine that I’d be sitting in the room [with clients] or watching [clients on] a TV screen. That felt like it could be way more immersive being there and for them too, to feel like they’re not alone in the process.

(Therapist 7)

Some therapists’ reactions to tele-VRET were neutral (16.7%, 3/18), reporting the need for more information or hands-on experience before forming an opinion.

What I’m not sure about is something like if the headsets are on and we’re in that space, something like spiders for example can [cause] a very physical reaction and people can flail. So if their headset comes off, what does that look like? Is there a protocol for that? Do they disappear? So that’s how I know they’re not there? And then I rejoin them in person or in video? Just being aware of how people respond. I don’t know because I’m not very familiar with virtual reality.

(Therapist 9)

Negative therapist reactions to tele-VRET (33.3%, 6/18) noted that stylized or nonphotorealistic avatars could break immersion or inhibit therapists’ ability to detect changes in affect (e.g., facial expressions) during an exposure session or therapy in general.

You kind of lost me on the avatar. I feel like the avatar takes it away from me as therapist, you as client, and makes it a character. I don’t want to necessarily be looking at an avatar of them. The avatar isn’t giving me a real indication of how they’re [feeling], what they’re looking [at], are they checked out? And you can’t get that with an avatar. I’ll hear them potentially talking, but I want to be able to see them. I want to be really connected with them at these points.

(Therapist 11)

### 3.7 Requested qualities and features of telehealth-based VRET

All therapists provided ideas about features and functions that could make tele-VRET helpful for clients and appealing for their practice.

#### 3.7.1 Tele-VRET for enhanced clinical capabilities

The majority of therapists (77.8%, 14/18) requested tele-VRET features to replicate or enhance clinical workflows for both exposure therapy and telehealth in general. These feature requests included ways to provide client-centered therapy during sessions, VR exercises for clients to complete between sessions, and allowing therapists to complete administrative tasks efficiently in VR.

[Building an exposure hierarchy] has always just been, brainstorm a list and then put them in order. I could imagine there’s a lot more [potential in VR], but the easiest thing could be if they brainstorm and you just write it all out, then you can move those into the hierarchy [in VR]…drag them over into an actual hierarchy that’s in order.

(Therapist 5)

Therapists described a wide range of VR features to facilitate their *in-session activities* for telehealth-based exposure therapy. These in-session VR features included interactive demonstrations of therapeutic exercises and 3D interfaces to co-create exposure hierarchies.

The option to demonstrate certain coping skills in the sessions might be something cool to have so a client can actually see how you do that. One that I’ll usually do is deep breathing. If you had a full body avatar, they could actually see the hand on the therapist’s stomach and chest rather than just if we’re doing a video session.

(Therapist 6)

What I’d love to be able to do is drag in this level, this activity, and then all the variations. It’s a lot of stuff. We’re going to go to a familiar restaurant with a loved one at a booth. And then, what about an unfamiliar restaurant without a loved one [not] at a booth? That level of detail. If we could then, in that environment, actually create the hierarchy together, “what would you rate this? What’s your predicted [Subjective Units of Distress Scale] for this?”

(Therapist 4)

Therapists also described how tele-VRET could allow for automated data collection to enhance the engagement and monitoring of *between-session exercises*.

I also wonder if there’s a record feature on it, so that while they’re doing it during homework, we could review it in our next session. Sometimes little things come up. They cough or they move [sideways]. Because the VR is so reactionary, that could affect the whole simulation that they’re in. Even just the audio that we could hear what’s going on, and potentially going back to the biofeedback loop to be able to see the heart rates when that’s happening.

(Therapist 11)

Therapists described complex exposure *workflows* and how it would be important for VR to reduce or add minimally to that complexity. Some therapists speculated that VR could function as a platform to unify tools and tasks.

I like the way that it looked integrated, that you could pull different things up. One of the things that comes up a lot for me [in exposure therapy over telehealth] is a lot of moving back and forth. We pull up white-boards for a while, and then we pull up a video, and then…from the [tele-VRET video] it appears to be very smooth and very straightforward. Rather than me being like, “ok, wait, hold on, I just have to find which of these 37 things I have open in my doc goes next.” So to me it feels like it might be more streamlined.

(Therapist 9)

However, therapists emphasized these features should be easy to use, should not interfere with their workflows, and should not introduce frustration for their clients.

The ease of use, user friendliness would be important. You don’t want to make things more complicated or more difficult for clients when they’re doing this kind of work because it’s already really difficult work. Anything that will keep that barrier to entry low is important. Whether it’s the actual headset, and ease of use, or us using it together, the actual experience…If it’s complicated and they’re distracted by those things, that’s actually going to interfere with the exposure.

(Therapist 16)

#### 3.7.2 VR content and customization

Most therapists (55.6%, 10/18) requested an expansive menu of preconfigured VR stimuli with options to customize stimuli creatively. Driving was the most commonly requested exposure stimulus but therapists also requested heights, flying, social situations, public places, injury or contamination, violence such as war or domestic abuse, and small animals such as snakes and spiders (Table 1). Therapists described how the process of finding an effective exposure was often surprising and unpredictable. To meet clients’ needs for remote exposure therapy, therapists emphasized the abilities to experiment with clients in VR, directly incorporate client feedback into VR arrangements, and retain those arrangements across sessions (e.g., persistent room configurations).

It’s about how I can adapt it to my client. My client might not have an issue with elevators. My client might have an issue with being in tight spaces, and the elevator will create that. My client might have an issue with spiders, not ants, but maybe the ant can evoke that kind of response as well. It’s about me as a clinician taking the [VR] curriculum and adapting to my clients in a way that works for them.

(Therapist 11)

[The most important thing is] the client’s ability to personalize the experience. There would have to be some large file of stock information that they can pull from or an option to upload your own. Have you ever played Minecraft? So they can build their own experiences. Or The Sims.

(Therapist 13)

### 3.8 Therapist concerns with tele-VRET

Therapists asked questions or expressed concerns about how tele-VRET might work in their practices. These concerns included client preferences and clinical contraindications, costs of VR in practice, VR safety and side effects, and the appropriateness of VR in imaginal exposure.

#### 3.8.1 Client preferences and contraindications

Most therapists (77.8%, 14/18) speculated that certain clients would be more receptive to tele-VR than others. Clients who are younger, experienced with technology, interested in video games, experience symptoms of OCD, or struggle with imaginal exposure were identified as leading candidates for tele-VRET. Therapists also described clients they considered ineligible for tele-VRET such as those who exhibit symptoms of psychosis, traumatic brain injury, migraines, or anxiety severe enough to be at risk for crisis or physical harm.

I think my younger adults are more confident with technology. They know what virtual reality is. I have a handful of young adults that loved a video game so it’s just an appealing thing to them. If you can find a way to already find something that they have excitement or passion about and incorporate it into their treatment, that’s always a good thing.

(Therapist 17)

They probably shouldn’t be doing [VR] if they have hallucinations and delusions. You don’t even have to have full-on schizophrenia. Somebody could be going through a really distressing time or maybe somebody who’s super high anxiety. They may not have the skill set to just take [the VR headset] off.

(Therapist 1)

#### 3.8.2 Costs

Half of the therapists (50%, 9/18) mentioned cost concerns; specifically, the costs to patients. Insurance coverage of VR equipment and services could be a dealbreaker as many clients relied on public health care or paid out of pocket for telemental health services.

Wanting it to be cost effective because then each client has to have one. So is that something then that insurance would cover as part of their treatment or did they have to pay for that out of pocket? I know I have a lot of clients that are on Medicare and disability. Financial concerns are always an issue…[using my VR in my office] might be the more cost effective way for some clients then.

(Therapist 17)

#### 3.8.3 Side effects and privacy of VR

Some therapists (22.2%, 4/18) expressed the need for thorough vetting of VR’s potential side effects (e.g., addiction) and compliance with privacy policies (e.g., HIPAA).

Your body can’t tell the difference and your brain can’t tell the difference. I worry about how [VR] can become like when video games came out. Our brain can get very attracted and addicted to something like that because it’s not reality where things are not perfect, right?

(Therapist 1)

#### 3.8.4 VR may be inappropriate for certain imaginal exposure techniques

An important minority of therapists (16.7%, 3/18) described how VR may be incompatible with some specific approaches to imaginal exposure therapy. These therapists provided imaginal exposure therapy for clients’ severe PTSD related to war or sexual abuse. Therapists described emphasizing clients’ own memories and interoceptive reactions rather than comfort during recreations of traumatic situations, and a general disagreement with the cartoonish appearance of VR.

The person’s memory of their own experience is obviously going to be the most evocative, right? Creating a [VR] cartoon of the thing you experienced would almost make it less intense…like it wouldn’t bring up as much emotion. I can’t see how the imaginal part would be improved with the virtual component. The memory lives in you and any way in which you alter it seems detrimental. Sometimes people will say, “I’m not even sure this really happened, but this is my memory.” The memory is what’s bothering you, so we’re still going to do exposure to what you think happened, even if it didn’t happen. It seems like I can’t think of a way of using VR for an imaginal. If you could, in theory, get it perfect, then that would be great. But you could never get it perfect because it happened 20 years ago.

(Therapist 10)

These are cartoons, these people are cartoons, these problems are cartoons.

(Therapist 3)

### 3.9 Opportunities of tele-VR in general

Therapists shared ideas for how they would like to use tele-VR for mental health practices other than exposure therapy. These other mental health therapies included biofeedback, roleplay therapy, gender identity exploration, and treatment of addiction.

Ten therapists (55.6%) were interested in *biofeedback* in tele-VR. These therapists emphasized the importance of teaching clients to recognize their own bodily responses as part of exposure therapy. Therapists described how VR environments could change in response to clients’ heart rate, breath rate, or galvanic skin response to train relaxation skills. Biofeedback could also be a clinically useful way to assess client affect, especially since VR avatars may not track facial expressions accurately.

I wonder if it couldn’t be programmed into the virtual reality to pick up on some of those nuances. If the client’s heart rate just dropped or their body basal temperature just went up, things like that. To reinforce that they have control over their own body responses, what works and what doesn’t work. VR can go a lot of places.

(Therapist 13)

If VR is really capturing the client’s movement, is there a way to get a reading on heart rate or any kind of [physiological measures]? If that can be incorporated, that would be golden because I would imagine little movements could throw you off. You’re not really getting that same feel of the facial expressions [in VR]. I would see a benefit in being able to get those readings of how somebody’s responding to something.

(Therapist 18)

Five therapists (27.8%) described how tele-VR could enhance their practice of *roleplay therapy*. Therapists reported difficulty directing or staging role play over telehealth, but imagined that immersion in VR could enhance those interactions when combined with customizable VR environments, avatars, and interactable objects. Therapists also speculated that nonplayer VR avatars could create more engaging empty chair therapy, allow clients to interact with copies of their own avatar for reflective roleplay, and how therapists could wear the avatar of another person to facilitate confrontational roleplay scenarios.

The [empty] chair represents your father who’s not here right now, and you’re going to talk to him. I might prompt the discussion and ask how they felt confronting their father about that thing that happened years ago. It’s just weird [in a video call] because they’re talking to me on a screen and they’re just looking to the left of me. But if you’re in this medaverse [sic], you could have an empty chair, you could have dad’s chair, you could have dad [his VR avatar].

(Therapist 11)

Two therapists (11.1%) described how customizable tele-VR avatars could help clients seeking therapy for *sexuality or gender identity*.

I have a client that has sexual orientation fears. They worry that they’re actually a lesbian. For now, their exposures are seeing imagery of heterosexual couples, but what would be cool with virtual reality is that we could position them with a man to where they might be touching or something like that. That, I think, would be something that they would never actually have the capacity to do in real life. But virtual reality could make that possible.

(Therapist 5)

I sometimes find myself working with people who have body dysmorphic disorder. In virtual reality, they can change [their avatars], which is really a tough thing because that’s what’s happening. They’re photo-shopping everything on Snapchat, then they look in the mirror and they don’t see that same [person]. So it may even help where they can see themselves as a different gender and function as a different gender and see if they can integrate that into who they are or if it is still a dissonance that they experience in a safe place without doing major changes. You know, hormone replacement, surgical changes. VR would be a nice start for that as well.

(Therapist 13)

One therapist (5.6%) expressed interest in tele-VR for addiction, particularly substance abuse. They described discussion in their organization to use VR to help clients navigate binge drinking in addiction-related situations (e.g., bars, parties, concerts).

We had talked about [VR for addiction therapy] at the hospital; having exposures where people go to a bar and have to be around virtual reality people and not drink. There’s drinking ones and certain drug ones and bars and parties and things like that. Raves or bands. And when you’re at a concert and stuff, like you could have certain ones set up for that too.

(Therapist 17)

## 4 Discussion

The goal of this study was to understand therapist perspectives on VR for telehealth-based exposure therapy. We interviewed 18 practicing telemental health therapists between October and December 2022. Most therapists had tried VR in the past and knew about VR for mental health care, but none had used VR in their own therapy services. Opinions about general VR were mostly positive with an equal amount of neutral and negative perceptions, the latter of which related to low perceived realism. After viewing a video demonstration of tele-VRET, therapists expressed interest in how VR could facilitate more immersive and interactive telehealth sessions and the importance of customizable places, objects, and situations for tele-VR therapy. Therapists requested a variety of specific tele-VRET situations such as driving, social interaction, violence, and small animals. Concerns about tele-VRET included client needs or preferences that might be incompatible with VR, costs for therapists and clients to access VR, side effects or risks to privacy, and uncertainty around VR for specific forms of imaginal exposure therapy. Therapists also discussed opportunities for tele-VR beyond exposure therapy, including biofeedback, roleplay, sex or gender identity, and addiction. Overall, these results demonstrate therapists’ interest in tele-VRET for clinical practice.

These findings contribute to the telemental healthcare literature in several important areas. First, while therapists reported broad benefits of telemental health services, they acknowledged telehealth involved communication limitations. These findings align with other research showing how conventional telemedicine modalities can obscure opportunities to connect with clients and build trust (Aviram and Nadan 2023; Cowan et al. 2019). Therapists in this study were enthusiastic about the potential to engage clients with VR technologies to build stronger therapeutic alliances. Second, therapists’ statements in this study broadly concurred with systematic reviews showing quantitative noninferiority of telehealth-based exposure therapy for PTSD (Olden et al. 2016; Scott et al. 2022). This study adds qualitative descriptions of some procedural adjustments therapists have made for conducting exposure therapy over telehealth. It is clear that therapists need technological solutions to enhance interactive sessions and closer therapeutic contact with clients (Mehraeen et al. 2023). Third, therapists in this study were broadly knowledgeable and interested in tele-VR, but expressed concerns over factors such as affordability, client access, ease of use, and therapeutic appropriateness. These results align closely with previous research on healthcare provider readiness to adopt therapeutic VR (Chung et al. 2022). In addition to identifying barriers and enablers of therapeutic VR, this study reports therapists’ requests for tele-VR features such as co-creating virtual spaces, automated data collection, and specific stimuli for use in tele-VRET. Collectively, these results signal abundant opportunities for multi-user VR therapy over telehealth (Meyerbröker and Morina 2021).

Therapists in this study expressed concerns about the implementation of VR into their telemental health practices. The most critical concern was that VR avatars could be perceived as unrealistic and inappropriate for use in clinical practice. There are many nuances to be explored in end-user preferences for tele-VR. Some research suggests simplistic and cartoonish VR avatars were perceived as comforting and trustworthy in the context of mental health therapy (Matsangidou et al. 2020). However, this preference for stylized VR avatars may be affected by factors other than esthetics. For example, VR avatars that are designed with cartoonish stylization can make it easier to avoid negative reactions related to the uncanny valley (Ma and Pan 2022). More research is needed to understand the relationships between VR avatar designs, provider perceptions, client preferences, and clinical outcomes. Another major concern was costs. Previous research showed that therapists did not view costs as a leading barrier to adoption of modern clinical VR (Lindner et al. 2019). However, costs were mentioned by 55.6% of therapists in this study, particularly for costs to clients. Therapists may still view costs as a barrier if they are unaware of the price and availability of modern VR equipment and software. This may signal the need for curation of low cost, accessible, and clinically relevant VR options (Sunkara et al. 2023).

Therapists provided novel ideas for tele-VR that can guide research for clinical implementation. Most of the requested features emphasized the need for therapists to creatively customize VR experiences for their clients. Demand for radically customizable VR therapy has been increasing in the literature (Baghaei et al. 2020; Halim et al. 2022), and some researchers have suggested VRET may lead to poor results if VR content does not match clients’ personal experiences or expectations (Chard et al. 2023). Emerging techniques for the nontechnical creation of self-produced VR content should be examined for clinical potential in the hands of therapists and their clients (Brandstätter 2023; Georgieva and Georgiev 2022; Hadjipanayi et al. 2023; Schöne et al. 2023). Therapists in this study described how the proposed features of tele-VRET could also expand clinical options beyond exposure therapy. While some therapists speculated that clients with dissociative disorders or schizophrenia might be poor candidates for tele-VR therapy, recent research has shown that VR may be an ideal platform to help these patients distinguish between reality and their symptomatic hallucinations (Bakk 2023; Bisso et al. 2020). Therapists were also enthused about the ability to interact more naturally with clients in VR than over conventional video-based telehealth, particularly in regard to therapeutic touch (Gallace and Girondini 2022). It may be beneficial to explore clinical applications and risks of VR-induced illusions of social body contact, which VR enthusiasts refer to as “phantom sense” (Fu et al. 2023; Piitulainen et al. 2022; Ramirez et al. 2023; Rasmus 2022).

These results should be interpreted in light of several limitations. Participants were recruited from the Doxy. me platform, which was also the employer of the research team. While the demographics of Doxy.me users have been consistent with overall mental healthcare industry statistics (American Psychological Association, 2022; Vogt et al. 2022; Wilczewski et al. 2022), future studies should aim to recruit from larger participant pools across a variety of telehealth networks. Relatedly, future studies would benefit from qualitative coding validation in collaboration with diverse stakeholder perspectives (O’Connor and Joffe 2020). Another limitation was that most therapists in this study had used VR in casual settings but none had used VR clinically. These therapists’ perspectives, then, were largely in response to the tele-VRET video and not based on direct hands-on experience. It is likely that therapists with experience in clinical VR would have different perspectives on tele-VRET. It will be vital for future research to obtain more diverse end-user perspectives, including mental health clients (Guillén et al. 2018). We presented descriptive percentages for context in this study, but the qualitative approach and small sample size means these percentages should not be viewed as representative. The qualitative insights generated in this study should be investigated quantitatively to obtain a better understanding of therapists’ perspectives on tele-VRET.

## 5 Conclusions

In conclusion, we found that experienced telemental health therapists had positive reactions to VR and creative ideas for clinical application of tele-VRET. An important minority of therapists expressed doubts about the perceived realism of VR, and there were general concerns about the costs and logistics of VR in practice. This cautious excitement will help inform the design and implementation of tele-VR for exposure therapy and other technological innovations in evidence-based mental health care.

## Supplementary Material

Supplementary

## Figures and Tables

**Table 1:** Specific objects, situations, and places requested for tele-VRET

Stimulus	Description	N (%)	Representative quote
Driving	Virtual simulations in which a client sits in or drives a car in busy traffic or down a quiet road	9 (50%)	“There’s an accident in front of you or you have to make a turn and then there’s a lot of oncoming traffic. You want the person to have that experience as much as possible. Or maybe that there’s like her [level] zero, which is just driving around a calm neighborhood and there’s no kid [running into traffic].” (Therapist 1)
Heights	Situations or places in which one could fall such as hiking, bridges, elevators, or staircases	6 (33.3%)	“Hiking and people falling. Like pushing people off of a higher ridge or an overpass or something.” (Therapist 5)
Flying	Being a passenger on a commercial airplane and precursor situations such as packing for a trip, traversing security lines, and boarding the plane	5 (27.8%)	“Flying is a great example. Most people don’t have the time or money to get on a flight every [session], or even go to an airport. It’s a big hassle for a lot of people and it’s just not realistic. So I think that’s a great example where [tele-VRET] could be really helpful.” (Therapist 16)
Social situations	Situations in which the client must interact with real or imagined others such as public speaking, job negotiations, classrooms, or parties	5 (27.8%)	“I could definitely see it being useful for social phobias, like people who are really afraid to go into groups to have conversations. Just practicing interaction and reading social cues and asking questions and managing your internal experience. I wonder if there could be an interesting virtual play space, because I do try to do exercises online but they worked better in person. Certain kinds of icebreakers and just being able to be a little more interactive and animated.” (Therapist 10)
Public places	Places that require leaving the home and entering uncontrolled spaces such as clinic offices, grocery stores, or restaurants	4 (22.2%)	“When people have anxiety related to trauma, where they feel safe and secure is really small. So exposure through VR could be really helpful. This is a coffee shop, this is a farmer’s market, this is a crowded grocery store, this is a graduation, concerts, ball games.” (Therapist 15)
Injury or contamination	The sight of another person or oneself bleeding, being bitten, or touching something unsanitary	4 (22.2%)	“Another client is [phobic of] rabies. She also has a fear of blood, so seeing a bandaid on the side of the road or seeing blood or having somebody bleeding, worried about contamination.” (Therapist 5)
Violence	Depictions of warzones or physical, sexual, or psychological abuse	4 (22.2%)	“I’ve got folks that have rape trauma. When you were talking about doing VR, I’m like, ‘I can’t in vivo that.’ So yeah.” (Therapist 7)
Small animals	The sight of and interaction with animals such as dogs, insects, snakes, or rats	4 (22.2%)	“The phobia of spiders or snakes or whatnot. I think that you could probably create pretty quickly.” (Therapist 17)
Enclosed spaces	Being in small and inescapable spaces such as elevators, car trunks, or trains	2 (11.1%)	“The very generalizable ones would be MRI or a train. They said it’s as if you’re at the very front of the train and watching.” (Therapist 15)
Swimming	Simulations of being near or in deep water	1 (5.6%)	“There’s gonna be a lot of different things they are afraid of. It can be something real that’s going on, like some person actually swimming.” (Therapist 2)
Disasters	Depictions of natural or manmade disasters such as storms or house fires	1 (5.6%)	“So a lot of the phobia stuff I feel like would be good. Like natural disasters. I’ve definitely worked with a number of people who’ve had house fires or things like that.” (Therapist 8)

VRET = virtual reality exposure therapy
