# Supplementary material for: Therapist perspectives on telehealth-based virtual reality exposure therapy
Source: Virtual Real. Author manuscript; Available in PMC 2024 Sep 5. (PMC11376200; doi:10.1007/s10055-024-00956-7)
Supplement: Supplementary [file NIHMS2018774-supplement-Supplementary.docx]

Appendix 1. Interview Guide

**Telemedicine for Mental Health Therapy**

“I’d like to talk about how you provide therapy. I’m interested in your own personal knowledge and experiences, so there are no right or wrong answers.”

A. “I’d like to start by learning about your mental health practice.”

A1. "What types of mental health services do you provide?"

A1a. “Which disorders do you mainly treat?”

A1b. “What age groups do you usually work with?”

A1c. “What type of therapies do you provide?”

A2. “How long have you been using telemedicine to provide therapy?”

A3. “In your experience, how is therapy different when provided over telemedicine *vs*. in-person?”

A3a. “What works well over telemedicine?”

A3b. “What aspects of therapy are more difficult over telemedicine?”

A3b1. “What kinds of distractions do you typically deal with during telemedicine sessions?”

B. “Great, thank you. I’m especially interested in learning about how you provide exposure therapy for phobias over telemedicine.”

B1. “How do you introduce the concept of exposure therapy to your clients over telemedicine?”

B1a. “Do you screen share visual materials?”

B1b. “Do you show informational videos?”

B1c. “Do you send them handouts?”

B2. “How do you go about planning exposure therapy with your clients over telemedicine?”

B2a. “How do you choose whether to use a graduated *vs*. flooding approach?”

B2b. “What do you use to create and manage exposure hierarchies? Word/Google Doc? Apps?”

B3. “What do your typical exposure therapy sessions look like over telemedicine?”

B3a. “What kinds of stimuli do you use for exposure therapy over telemedicine?”

B3a1. “Do you use pictures?”

B3a2. “Videos?”

B3a3. “Real life objects or situations?”

B3b. “How do you assess your clients’ anxiety levels during exposure therapy exercises?”

B3b1. “What scale do you have your clients use to measure their fear or anxiety during exposure exercises in-session (e.g., like a 0-10 scale)?”

B3b2. “How often do you assess their fear or anxiety during exposure exercises in-session (e.g., every 10 minutes; before, at peak, after)?”

B3c. “How do you decide when to move to the next step in the exposure hierarchy?”

B3d. “How do you decide when to end the exposure therapy exercise for the session?”

B4. “Do you ask clients to practice exposure exercises between telemedicine sessions? If so, how does that typically look?”

B4a. “How do you assign between-session exposure exercises? Verbally? Via email?”

B4b. “How often do you ask clients to practice exposure exercises between sessions?”

B4b1. “Once a week? Daily? Multiple times per day?”

B4c. “How long do you ask clients to remain in feared situations when practicing exposure exercises between sessions?”

B4d. “What kinds of stimuli do you ask your clients to use between-session exposure exercises?”

B4d1. “Do you use pictures?”

B4d2. “Videos?”

B4d3. “Real life objects or situations?”

B4e. “How do you assess your clients’ anxiety levels when they do exposure exercises between-sessions?”

B4e1. “What scale do you have your clients use to measure their fear or anxiety during between-session exposure exercises (e.g., like a 0-10 scale)?”

B4e2. “How often do you have your clients record their fear or anxiety when (e.g., every 10 minutes; before, at peak, after)?”

B4e3. “How do your clients record their anxiety levels and provide those data to you?”

B4f. “How do you track client completion of between-session exposure exercises?”

**Virtual Reality**

C. “I’d like to spend the last half of the interview talking about virtual reality.”

C1. “How would you define or describe virtual reality?”

C2. “What experiences have you had with virtual reality?”

C3. “What are your overall impressions about virtual reality?”

C4. “What have you heard about virtual reality for mental health therapy, particularly exposure therapy?”

C5. “Have you used virtual reality in your therapy practice? Why or why not?”

D. “We’re developing a telemedicine-based, virtual reality clinic for therapists and clients to meet remotely. Virtual reality uses headsets to place a display over your eyes and speakers near your ears to fully immerse you in simulated environments. Numerous randomized control trials have shown exposure therapy in virtual reality is just as effective as in-person. Exposure therapy in virtual reality can also be easier since the feared objects or situations are programmed into the headset instead of managed by therapists in the clinic. I’d like to play a brief video to show an example of what we’re planning to develop. [show video]. Now I’d like to get your impressions about this proposed virtual reality clinic. As a reminder, there are no right or wrong answers, and we are interested in your genuine opinions.”

D1. “What are your initial thoughts on this telemedicine-based virtual reality clinic?”

D1a. “What would you think of using this virtual reality clinic with your telemedicine clients?”

D1b. “How well would this virtual reality clinic fit into your telemedicine workflow?”

D2. “What should we focus on to support therapists using this virtual reality clinic to provide exposure therapy?”

D2a. “What features should we include to help therapists introduce the concept of exposure therapy to clients?”

D2b. “How can we build tools to help therapists create and manage exposure hierarchies in this virtual reality clinic?”

D2c. “What features would be useful for therapists to manage stimuli or simulated environments in this virtual reality clinic?”

D2d. “What would be good ways for us to help therapists assess and record client anxiety during virtual reality exposure therapy exercises?”

D2d1. “How do you think therapists would prefer to receive those data?”

D2e. “Would it be helpful if clients could use this virtual reality clinic to practice exposure exercises between therapy sessions?”

D2e1. “How can we make it easy for therapists to assign virtual reality exposure exercises between therapy sessions?”

D2f. “What other therapeutic activities or features would you like to see in this virtual reality clinic?”

D3. “What information would help you decide if this virtual reality clinic was worth using in your therapy practice?”

D3a. “How important is word of mouth from other therapists?”

D3b. “How important is clinical evidence from research studies?”

D3c. “How important are requests from clients?”

D3d. “How important are privacy and HIPAA in virtual reality therapy?”

D4. “What kind of training would you want before using this virtual reality clinic to conduct telemedicine-based exposure therapy?”

D5. “What concerns, if any, would you have about using this virtual reality clinic with clients?”

D6. “How do you think clients would react to receiving exposure therapy using this virtual reality clinic?”

D6a. “How confident would they feel using the new technology?”

D6b. “What kind of prior training would you want for your clients?”

D6c. “Which clients would you recommend most for therapy using this virtual reality clinic? Why?
